# sLORETA intracortical lagged coherence during breath counting in meditation-naïve participants

**DOI:** 10.3389/fnhum.2014.00303

**Published:** 2014-05-15

**Authors:** Patricia Milz, Pascal L. Faber, Dietrich Lehmann, Kieko Kochi, Roberto D. Pascual-Marqui

**Affiliations:** Department of Psychiatry, Psychotherapy and Psychosomatics, The KEY Institute for Brain-Mind Research, University Hospital of PsychiatryZurich, Switzerland

**Keywords:** sLORETA, breath counting, meditation, lagged coherence, functional connectivity, EEG, intracortical coherence

## Abstract

We investigated brain functional connectivity comparing no-task resting to breath counting (a meditation exercise but given as task without referring to meditation). Functional connectivity computed as EEG coherence between head-surface data suffers from localization ambiguity, reference dependence, and overestimation due to volume conduction. Lagged coherence between intracortical model sources addresses these criticisms. With this analysis approach, experienced meditators reportedly showed reduced coherence during meditation, meditation-naïve participants have not yet been investigated. 58-channel EEG from 23 healthy, right-handed, meditation-naïve males during resting [3 runs] and breath counting [2 runs] was computed into sLORETA time series of intracortical electrical activity in 19 regions of interest (ROI) corresponding to the cortex underlying 19 scalp electrode sites, for each of the eight independent EEG frequency bands covering 1.5–44 Hz. Intracortical lagged coherences and head-surface conventional coherences were computed between the 19 regions/sites. During breath counting compared to resting, paired *t*-tests corrected for multiple testing revealed four significantly lower intracortical lagged coherences, but four significantly higher head-surface conventional coherences. Lowered intracortical lagged coherences involved left BA 10 and right BAs 3, 10, 17, 40. In conclusion, intracortical lagged coherence can yield results that are inverted to those of head-surface conventional coherence. The lowered functional connectivity between cognitive control areas and sensory perception areas during meditation-type breath counting compared to resting conceivably reflects the attention to a bodily percept without cognitive reasoning. The reductions in functional connectivity were similar but not as widespread as the reductions reported during meditation in experienced meditators.

## Introduction

The ever-changing functional states of the brain are constituted by the activity of spatially distributed neural networks (Mesulam, 1990; Tononi et al., 1998; Fuster, 2006) that involve interactions between brain regions. The electric mechanisms of these interactions have been assessed with various measures of brain electric (EEG) functional connectivity (e.g., David and Friston, 2003; Kelly et al., 2003; Pascual-Marqui, 2007; Srinivasan et al., 2007; Stam et al., 2007). Measures of functional connectivity for the assessment of brain states lately have received much attention. Several connectivity studies described characteristic changes between various mental states (e.g., Stam, 2000; Burgess and Ali, 2002; Mizuhara et al., 2005; White et al., 2009).

A mental state of prominent current interest in neuroscience is meditation (Davidson et al., 2003; Lutz et al., 2004, 2009; Vaitl et al., 2005; Cahn and Polich, 2006; Slagter et al., 2007; Van Den Hurk et al., 2010; Travis, 2011; Dickenson et al., 2013). Meditative states reportedly differ from task-free resting states in functional EEG connectivity. Increases as well as decreases of EEG functional connectivity during meditation compared to resting have been reported for several EEG frequency bands: increases and decreases in theta (Aftanas and Golocheikine, 2001), increases in alpha (Levine, 1976; Dillbeck and Bronson, 1981; Travis and Wallace, 1999; Travis, 2001), and increases in gamma (Lutz et al., 2004). The utilized different EEG references, the different analysis approaches and the different participant groups (i.e., meditation traditions, meditation experience) may account for the differing results.

These results were all obtained by computing functional connectivity between head-surface-recorded time series. Over the past years, criticism of such connectivity results has increased, observing that their interpretation is severely limited because of (1) the ambiguity of localization since not all brain electric sources are radially oriented toward the head surface, (2) dependence on the chosen reference, and (3) volume conduction that produces non-physiological values with zero phase lag between data time series (Nolte et al., 2004; Ruchkin, 2005). Computing functional connectivity as coherence via intracortical source modeling (e.g., via LORETA, Pascual-Marqui, 2002; Lehmann et al., 2006) addresses problems (1) and (2), and excluding coherences with zero phase lag (e.g., using “lagged coherence,” Pascual-Marqui, 2007) addresses problem (3). Lagged coherence excludes the non-lagged part of coherence which comprises effects of volume conduction (artificially increasing coherence), and effects of non-recorded sources that simultaneously drive recorded sources (conceivable, but requiring very special circumstances).

Using the suggested analysis approach of “intracortical lagged coherence,” experienced meditators of five different traditions were studied during resting and meditation; only decreases of coherence were seen during meditation in eight EEG frequency bands covering 1.5–44 Hz (Lehmann et al., 2012).

Several studies report that meditation practice has long-term effects on the functioning of neuronal networks (Tebecis, 1975; Newberg et al., 2001; Lutz et al., 2004; Aftanas and Golosheykin, 2005; Lazar et al., 2005; Tei et al., 2009; Saggar et al., 2012). In order to elucidate the neural mechanisms of meditating as such while excluding confounding effects of meditation experience and associated trait changes, it is crucial to investigate meditation in participants who are not trained in meditation (Dickenson et al., 2013). We further suggest that participants should not be aware that they are performing a meditative exercise (“meditation-unaware” participants) because participants who view themselves as novice meditators might experience strong frustration when realizing difficulties in reaching a state they might feel obliged to attain (Aftanas and Golocheikine, 2001), thus introducing a confounding factor for brain electric activity.

Functional connectivity during meditation has not yet been investigated in meditation-unaware, non-trained participants. Attention to one's breath reportedly can induce a meditative state even in non-trained participants (Dickenson et al., 2013). Thus, not surprisingly, common to different meditation traditions is that meditative practice is initiated with the exercise of “breath counting” (Shapiro, 1980; Walshe, 1987; Kubota et al., 2001; Murata et al., 2004; Takahashi et al., 2005). Breath counting is a simple task which solely requires the silent counting of exhalations during sustained awareness of the autonomous bodily process of breathing.

Our study protocol took variations of task performance into account, because—although breath counting is a seemingly simple task—unintended intruding thoughts are common during task performances (Kubose, 1976; Uleman and Bargh, 1989). Subjective reports about experienced quality of performance were useful for the analysis of EEG during attention demanding tasks (Berka et al., 2007) and meditative states in particular (Braboszcz and Delorme, 2011). In general, the value of first-person perspective in brain activity analyses has recently been stressed (Lutz et al., 2002; Jack and Roepstorff, 2003; Overgaard et al., 2008; Qin et al., 2011). Accordingly, our participants did the breath counting exercise twice and ranked the subjective quality of their task performance after the EEG recording. The better run was used for analysis.

The present study examined intracortical lagged EEG coherence during no-task resting and breath counting in non-trained, meditation-unaware participants, and related the results to our meditation study (Lehmann et al., 2012). To compare with other earlier studies, conventional head-surface EEG coherence was also computed.

We hypothesized that breath counting in meditation non-trained and meditation-unaware persons leads to decreased functional connectivity compared to resting.

## Materials and methods

### Participants

Healthy right-handed male volunteers were invited via flyers at the Swiss Federal Institute of Technology Zurich. Applicants were screened during a structured telephone interview. Only male participants were considered because of the effects of menstrual cycle and hormonal contraceptives on EEG in women (Creutzfeldt et al., 1976; Becker et al., 1980, 1982; Krug et al., 1999). Previous experience with meditation, history of head trauma or brain disease and current drug usage were exclusion criteria. The first 25 qualifying men were invited to participate in the EEG recordings. The Ethics Committee of the Canton of Zurich approved the experimental protocol. All participants gave their written informed consent. Breath counting was introduced as an attention exercise, and its relation to meditation was not mentioned until after the experiment. Each participant received 30 Swiss Francs. Eventually, after EEG pre-processing, data from 23 participants were available (mean age 23.2 years, *S.D*. = 1.9, range = 19–27).

Characteristics of the participants such as education, occupation, and individual skills (sport, art, music, or prayer) may possibly relate to their ability for internal concentration. In our sample these were as shown in Table 1. We note that none of the participants was a professional in any sports, music or artistic domain.

**Table 1:** **Personal characteristics of the participants**.

	**Sports**			**Music/art**			**Praying**
**Semester**	**Type(s)**	**Frequency**	**Duration**	**Type(s)**	**Frequency**	**Duration**	**Frequency**
2	–	–	–	–	–	–	–
2	–	–	–	Maths	Daily	>1 h	–
2	Gym, biking, walking	Few times a week	>1 h	Guitar	Daily	>1 h	–
2	–	–	–	Piano	Few times a week	15–45 min	–
2	–	–	–	–	–	–	–
2	–	–	–	–	–	–	–
4	–	–	–	–	–	–	–
4	Biking, gym, jogging	Daily	>1 h	–	–	–	–
5	Walking, jogging, boxing	Few times a week	>1 h	Painting, dancing	Few times a week	>1 h	–
6	Biking	Daily	15–45 min	Piano, harp	Weekly	>1 h	–
6	Jogging	Weekly	>1 h	–	–	–	–
8	Jogging, volleyball	Few times a week	>1 h	–	–	–	–
8	Walking	Less than weekly	>1 h	–	–	–	–
8	Hockey	Few times a week	>1 h	–	–	–	–
9	–	–	–	Guitar, piano	Few times a week	15–45 min	–
9	Diving	Few times a week	>1 h	Piano	Few times a week	15–45 min	–
9	Gym	Few times a week	>1 h	–	–	–	–
10	–	–	–	Singing, piano	Few times a week	15–45 min	Regularly
10	Swimming, jogging, dancing	Few times a week	15–45 min	–	–	–	–
10	–	–	–	–	–	–	–
10	Squash	Few times a week	15–45 min	–	–	–	Regularly
11	Tennis, gym, hockey	Few times a week	>1 h	Guitar	Less than weekly	15–45 min	–
12	Jogging, swimming	Few times a week	15–45 min	–	–	–	–

The NEO-FFI (Borkenau and Ostendorf, 1993) neuroticism personality scores of the participants were: mean = 14.9, *S.D*. = 6.2, extraversion: mean = 26.5, *S.D*. = 6.8, openness: mean = 33.0, *S.D*. = 6.7, agreeableness: mean = 31.3, *S.D*. = 5.70 and conscientiousness: mean = 31.7, *S.D*. = 6.5.

### Procedure

During electrode attachment (Easy-Cap, Munich, Germany), the breath counting task was verbally explained and participants were informed that during each of the upcoming recording runs they should sit relaxed with arms and legs in a comfortable position. Participants were then seated in an electrically, acoustically and light-shielded chamber on an armchair, viewing a computer monitor at 1 m. Each participant was recorded during three resting runs and two breath counting runs (each run 5 min) in the sequence resting, breath counting, resting, breath counting, resting.

Instructions were presented on the monitor prior to each run. Before resting runs, participants were asked to think freely of whatever they wanted, and to close their eyes until a tone would indicate the end of the run. Before breath counting runs, participants were asked to pay attention to their breathing, to breathe through their nose, and to silently count their exhalations up to 10 and then to start again from 1. They should also start again from 1 if they lost track of their count. The participants were also asked not to breathe according to their counting but count according to their breathing, and to let intervening thoughts pass by and concentrate on respiration. The participants were asked to concentrate completely on the task and to try not to digress. The instructions for all runs finished by asking participants to close their eyes, start task execution and maintain it until a tone would indicate the end of the run.

After the end of all recordings, the participants were asked during which of the two breath counting runs they subjectively felt they had performed better. This “better breath counting run” was the first run in 9 and the second run in 14 participants.

The participants were also asked to describe how they did their breath counting. The answers included attention to the percept in the nostril, to the movement of their abdomen, or to the sound of their breath.

Participants eventually were asked whether they had ever before done the breath counting exercise and whether they were aware of a relation between breath counting and meditation. All participants answered no to both questions.

### Recording

EEG data were recorded using a 64-channel EEG system (M&I, Prague, Czech Republic) with 0.05–128 Hz band pass, digitized at 250 samples/s. Fifty eight electrodes were placed according to the international 10–10 system (Chatrian et al., 1985; Nuwer, 1987) omitting C1 and C2. Recording reference was Cz. Eye movements were recorded by two additional electrodes above the right and below the left eye, referenced to Fpz.

### Data preprocessing

The EEG data was pre-processed using BrainVision Analyzer 2 (Brain Products, Munich, Germany). Channels with severe artifacts (mean across participants = 1.6, *S.D*. = 2.0) were replaced using the analysis software's spline interpolation. Independent component analysis was used to remove eye movement artifacts. Sweat, muscle, movement and electrode artifacts were excluded based on visual inspection of the screen display of the entire recordings. The data were segmented into data epochs of 2 s, re-referenced to average reference, and up-sampled to 256 Hz using spline interpolation. If for a given participant, fewer than 10 data epochs were available in anyone of the five runs, the participant was omitted (2 of 25 participants). Eventually, on average per participant, the mean number of available data epochs from the five runs ranged between 107 and 118 (*S.D*. between 23 and 30).

### Power spectral analysis

For each participant, data epoch and channel, FFT was used to compute the power spectrum. The spectra were averaged across epochs for each recording run, and averaged across the 58 channels. Integrated spectral power was computed for the eight independent EEG frequency bands (Kubicki et al., 1979; Niedermeyer and Lopes Da Silva, 2005): delta (1.5–6 Hz), theta (6.5–8 Hz), alpha-1 (8.5–10 Hz), alpha-2: (10.5–12 Hz), beta-1 (12.5–18 Hz), beta-2 (18.5–21 Hz), beta-3 (21.5–30 Hz), and in addition, gamma (35–44 Hz). Values of the three resting runs were averaged. Paired *t*-tests compared log-transformed band power between averaged resting runs and the better breath counting run, separately for the eight EEG frequency bands (*p* < 0.05 are reported without correction for multiple testing).

Additionally, paired *t*-tests compared log-transformed band power between averaged resting runs and the better breath counting run separately for the 58 channels and for each of the eight EEG frequency bands.

### sLORETA current density

From 58-channel EEG, intracortical current density at 6239 voxels was computed using standardized low resolution electromagnetic tomography (sLORETA, Pascual-Marqui, 2002) as implemented in the software package available at: http://www.uzh.ch/keyinst/loreta. sLORETA allows the estimation of recordings of intracortical signals, which can be informally described as corresponding to virtual, non-invasive, intracortical electrodes. The sLORETA analysis was done separately for each of the eight independent EEG frequency bands defined above.

Paired *t*-tests compared sLORETA current density between the averaged resting runs and the better breath counting run for the 19 ROIs (Regions of Interest) defined for sLORETA-based intracortical lagged coherence computation as described below, separately for the eight EEG frequency bands.

### sLORETA-based intracortical lagged coherence

Brain connectivity analysis in this study used two methodological developments that depart fundamentally from previous literature. Firstly, estimated signals of intracranial electric neuronal activity were analyzed, rather than head-surface EEG signals of electric potential differences. Secondly, physiological measures of lagged connectivity were used, instead of classical coherence-type measures that mostly indicate common sources and not true connectivity.

Formally, let **X**_*i*_(*t*), **Y***_i_(t)* ∈ ℝ^3× 1^ denote the three dimensional time series for the current density vectors (i.e., the intracranial signals of electric neuronal activity) at any two voxels, for the *i*-th recording epoch, at time “*t*.” The complex-valued Fourier transforms at frequency ω are denoted **X**_*i*_(ω), **Y**_*i*_(ω) ∈ ℂ^3×1^. For *N* recording epochs, the complex-valued covariance can be written in partitioned form as:

$$S=\left(\begin{array}{cc} S_{YY} & S_{YX}\\ S_{XY} & S_{XX} \end{array}\right)=\frac{1}{N}\sum_{i=1}^{N}Z_{i}\left(\omega \right)\;Z_{i}^{*}\left(\omega \right)$$

with:

$$Z_{i}\left(\omega \right)=\left(\begin{array}{c} X_{i}\left(\omega \right)\\ Y_{i}\left(\omega \right) \end{array}\right)$$

and where the superscript “*” denotes the transposed and complex conjugate vector. The partitioned coherence matrix which conserves each intra-voxel structure is:

$$R=\left(\begin{array}{cc} S_{YY}^{-1/2} & 0\\ 0 & S_{XX}^{-1/2} \end{array}\right)\left(\begin{array}{cc} S_{YY} & S_{YX}\\ S_{XY} & S_{XX} \end{array}\right)\left(\begin{array}{cc} S_{YY}^{-1/2} & 0\\ 0 & S_{XX}^{-1/2} \end{array}\right)$$

Finally, as explained in detail in Pascual-Marqui et al. (2011), the difference between the appropriate statistics for testing total and instantaneous connectivities gives the well-defined measure of lagged connectivity:

$$F_{X⇄Y}=\ln\left|\text{Re}\left(R\right)\right|-\ln\left|R\right|$$

or its transformation as lagged coherence:

$$r_{lag}^{2}=1-\exp\left(-F_{lag}\right)$$

In the particular case for univariate time series *x_i_(t)* and *y_i_(t)*, the lagged coherence has a very simple and appealing form:

$$r_{lag}^{2}=\frac{{\left[s_{xy}^{\text{Im}}\left(\omega \right)\right]}^{2}}{s_{xx}\left(\omega \right)s_{yy}\left(\omega \right)-{\left[s_{xy}^{\text{Re}}\left(\omega \right)\right]}^{2}}$$

where the superscripts “Re” and “Im” denote real and imaginary parts, respectively.

It is shown in Pascual-Marqui (2007) and Pascual-Marqui et al. (2011) that the lagged connectivity measure for intracranial signals contains physiological information minimally affected by volume conduction artifacts. Furthermore, note that while coherence quantifies the linear relationship between complex-valued variables, lagged coherence measures exactly the same, but with the exclusion of zero-lag contribution.

19,459,441 connectivities can be computed between all pairs of the 6239 voxels. In order to keep the results in a manageable range, we parsed the entire LORETA result space of 6239 voxels into a limited number of ROIs. Unfortunately, there are no rules for determining ROIs. In this study, in order to explore interconnectivity between all major brain areas, the cortex underlying the 19 standard electrodes (Fp1/2, F7/8, F3/4, Fz, T7/8, C3/4, Cz, P7/9, P3/4, Pz, O1/2) of the 10/20 system (Jasper, 1958) was used for the definition of the ROIs. These cortical areas are well documented in other low resolution tomographies such as NIRS (Jurcak et al., 2007). The radius around each ROI was chosen at 20 mm. The current density values of all voxels within the ROI were averaged. Using the sLORETA intracortical current density time series of these 19 ROIs, intracortical lagged coherences (Pascual-Marqui, 2007) were computed for all pairs of ROIs.

For each participant, sLORETA intracortical lagged coherence (sLORETA software option “linear lagged connectivity”) was analyzed in that breath counting run for which participants *post-hoc* had reported better subjective performance; this run will hereafter be called “breath counting.” There was no significant difference of the lagged coherences between the three resting runs. To minimize the influence of time, the sLORETA intracortical lagged coherence values of the three resting runs were averaged; this average hereafter will be called “resting.”

Using paired t-statistics, sLORETA intracortical lagged coherence was compared between breath counting and resting.

The 19 ROIs yield 171 (= 19^*^18/2) distinct coherences and therefore allow 171 paired *t*-tests between conditions for each of the eight independent EEG frequency bands. *P*-values were corrected for multiple testing.

In order to check whether the coherences whose significance withstood correction for multiple testing represent the general trend of the results, the number of significantly higher and lower coherences was determined for each frequency band, reducing statistical thresholding to uncorrected *p* < 0.01. The significance of the observed ratio was obtained using Yates-corrected chi-square values. This procedure can be regarded as a procedure analogous to an exceedence proportion test (Friston et al., 1990, 1991).

### Head-surface conventional coherence

Conventional squared coherence (sLORETA software option “linear total connectivity”) was computed from the head-surface recorded data for the same eight frequency bands and between the 19 electrodes of the international 10–20 system for the same data sets as described in the preceding section.

Following the previous notation, this measure is defined as:

$$r_{Total}^{2}=\frac{{\left[s_{ab}^{\text{Re}}\left(\omega \right)\right]}^{2}+{\left[s_{ab}^{\text{Im}}\left(\omega \right)\right]}^{2}}{s_{aa}\left(\omega \right)s_{bb}\left(\omega \right)}$$

where the subscripts “*a*” and “*b*” denote two head-surface EEG signals. It has been shown in Nolte et al. (2004), Pascual-Marqui (2007), and Pascual-Marqui et al. (2011) that this measure is very little related to true physiological connectivity, and that it is mostly determined by common sources that affect instantaneously via volume conduction all scalp EEG electrodes. In particular, calculations performed in Pascual-Marqui (1993) show an enlightening example where the head-surface total coherence can reach values as high as 0.83 in a hypothetical brain that is totally disconnected. The reason for showing these non-physiological, mostly artifactual results based on head-surface-based conventional coherence is to allow a comparison to previously published related results.

### Significance correction for multiple testing

Where needed, band-wise correction for multiple testing was calculated with the non-parametric randomization methodology (Nichols and Holmes, 2002) implemented in the sLORETA software package. Non-parametric randomization for the multivariate case, e.g., for all voxels and frequency bands, or for all pairs of voxels and frequency bands, uses randomization for the estimation of the empirical probability distribution under the null hypothesis being tested. This procedure is applied to the “maximum-statistics,” which makes the best possible empirical adjustment to the multivariate covariance structure, thus correcting for multiple testing in a much improved way as compared to, for instance, Bonferroni's correction.

## Results

### Power spectral analysis

Average head-surface EEG spectral power across participants was higher for breath counting compared to resting for all frequency bands except for the alpha-1 band. However, none of the differences reached *p* < 0.05 (no correction for multiple testing).

In the channel-wise 464 tests (58 separate channels for each of the eight EEG frequency bands), only one channel (F1) in alpha-1 showed significantly higher power during breath counting than averaged resting [*t* = 3.02, *p*(corrected for multiple testing) = 0.036].

### sLORETA current density

sLORETA current density was higher during breath counting than resting in alpha-2 and beta-2 through gamma EEG frequency bands, lower in delta through alpha-1 and beta-1. However, in the ROI-wise 152 tests (19 separate ROIs for each of the eight EEG frequency bands), none of these differences reached *p*(corrected for multiple testing) <0.05.

### sLORETA-based intracortical lagged coherence

**Figure 1:**
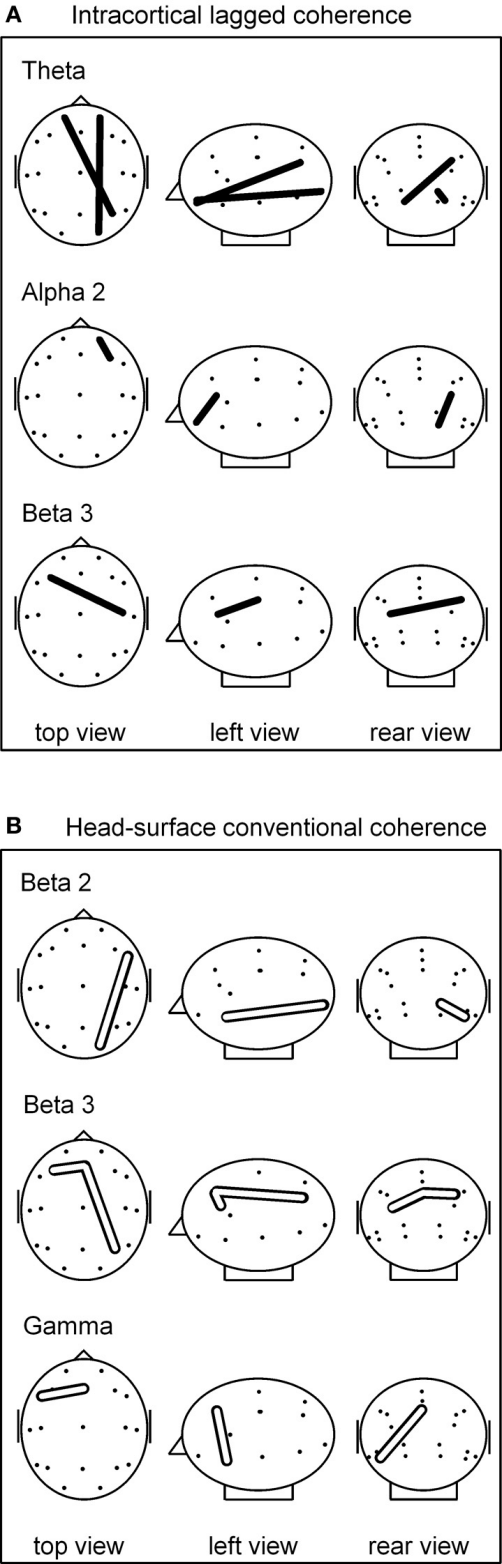
**(A)** sLORETA-based intracortical lagged coherences that differed during breath counting compared to resting at *p*(corrected) < 0.05. All four connectivities were lower during breath counting. **(B)** Head-surface conventional coherences that differed during breath counting compared to resting at *p*(corrected) < 0.05. All four connectivities were higher during breath counting. Glass brain axial, sagittal, and coronal views, left ear left; ROI locations are indicated by dots. For ease of visualization, the intracortical ROIs are represented by the corresponding head-surface locations of the standard 19 electrode positions of the international 10–20 system.

Comparing breath counting to resting, four differences of sLORETA-based intracortical lagged coherences between conditions were significant at *p* < 0.05 after correction for multiple testing; all four had lower values during breath counting. The results are listed in the upper part of Table 2A that includes the magnitudes of coherences, *t*-values and uncorrected *p*-values, and are illustrated in Figure 1A.

**Table 2:** **(A) Coherences that differed between breath counting (BC) and resting (R) at *p*(corrected) < 0.05. Intracortical lagged coherences (top), and head-surface conventional coherences (bottom). (B) Head surface coherences between electrode sites corresponding to the ROIs in a (top), and intracortical lagged coherences between ROIs corresponding to the electrode sites in a (bottom)**.

	**(A) Sites at *p* (cmt) < 0.05**										**(B) Corresponding sites**								
	**Intracortical lagged coherence**										**Corresponding head-surface coherence**								
	**From**		**To**		**Coherence**						**From**		**To**		**Coherence**				
**Hz-Band**	**BA**		**BA**		**BC**	***R***	**BC-R**	***t***	***p***	***p* (cmt)**	**Electrode**				**BC**	**R**	**BC-R**	***t***	***p***
T	10	L	40	R	0.154	0.191	**–0.037**	**–3.91**	0.001	0.04	Fp1	L	P4	R	0.611	0.601	0.010	2.10	0.05
T	10	R	17	R	0.163	0.191	**–0.027**	**–4.18**	0.0004	0.02	Fp2	R	O2	R	0.564	0.560	0.004	0.76	0.5
A2	10	R	10	R	0.193	0.227	**–0.034**	**–4.32**	0.0003	0.01	Fp2	R	F4	R	0.748	0.748	0.000	0.14	0.9
B3	10	L	3	R	0.071	0.084	**–0.013**	**–4.26**	0.0003	0.02	F3	L	C4	R	0.207	0.212	**–0.005**	**–0.79**	0.4
	**Head-surface conventional coherence**										**Corresponding intracortical lagged coherence**								
	**From**		**To**			**Coherence**					**From**		**To**		**Coherence**				
**Hz-Band**	**Electrode**				**BC**	***R***	**BC-R**	***t***	***p***	***p* (cmt)**	**BA**		**BA**		**BC**	***R***	**BC-R**	***t***	***p***
B2	F8	R	O2	R	0.390	0.364	0.026	4.21	0.0004	0.03	47	R	17	R	0.137	0.144	**–0.007**	**–0.99**	0.3
B3	F3	L	Fz	M	0.607	0.592	0.015	4.26	0.0003	0.02	10	L	8	M	0.105	0.095	0.010	1.33	0.2
B3	Fz	M	P4	R	0.441	0.425	0.016	3.91	0.001	0.04	8	M	40	R	0.084	0.089	**–0.006**	**–2.46**	0.02
G	F7	L	Fz	M	0.285	0.247	0.038	3.57	0.002	0.05	47	L	8	M	0.071	0.077	**–0.007**	**–1.20**	0.2

The significantly differing, lower coherences comprised two cases in the theta frequency band, and one each in the alpha-2 and in the beta-3 band. All involved ROIs in BA 10 (middle frontal gyrus). In the theta band, coherences were lower between left BA 10 and right BA 40 (inferior parietal lobule), and between right BA 10 and right BA 17 (cuneus). In the alpha-2 band, a short-distance coherence was lower within right BA 10. In the beta-3 band, coherence was lower between left BA 10 and right BA 3 (postcentral gyrus).

Reducing the statistical thresholding showed that the four significantly changed coherences reported above represent a general trend: At uncorrected *p* < 0.01, 26 coherences were lower during breath counting than resting and only one was higher (upper Table 3). This ratio of 26 cases of decreased vs. 1 of increased coherence significantly deviates from chance at chi-square = 23.15, *p* < 0.0000015.

**Table 3:** **Number of coherences higher (>) or lower (<) during breath counting (BC) than resting (R) at *p* < 0.01 in the eight EEG frequency bands**.

**Hz-Band**	**D**	**T**	**A1**	**A2**	**B1**	**B2**	**B3**	**G**	**Sum**
	**Intracortical lagged coherences**								
BC > R							1		1
BC < R	5	6	3	4		3	3	2	26
	**Head-surface conventional coherences**								
BC > R	4					3	7	3	17
BC < R		1			4	1		1	7

*Top: Intracortical lagged coherences. Bottom: Head-surface conventional coherences. Hz-Band, EEG frequency band; D, delta; T, theta; A1, alphsa-1; A2, alpha-2; B1, beta-1; B2, beta-2; B3, beta-3; G, gamma*.

### Head-surface conventional coherence

Comparing the breath counting to resting, four differences of head-surface conventional coherences between the breath counting and resting condition were significant at *p* < 0.05 after correction for multiple testing; all four had higher values during breath counting, thus showed differences contrary to those observed with intracortical lagged coherences. The results are listed in the lower part of Table 2A and are illustrated in Figure 1B. The significantly differing, higher coherences comprised one case in the beta-2 band (over the right side of the head), two cases in the beta-3 (frontal midline to the left and to the right), and one in the gamma band (within left anterior region).

Reducing the statistical thresholding showed that the four significantly changed coherences reported above represent a general trend: At uncorrected *p* < 0.01, 17 coherences were higher during breath counting than resting and seven were lower (lower Table 3). This ratio of 17 cases of increased vs. 7 of decreased coherences significantly deviates from chance at chi-square = 4.17, *p* = 0.041.

### Comparing intracortical lagged coherence to head-surface conventional coherence

The coherences that significantly differed between conditions were much lower for intracortical lagged coherences than for head-surface conventional coherences (Table 2), with ranges of 0.071–0.193 and 0.247–0.607, respectively.

In the four EEG frequency bands where head-surface coherence was significantly higher during breath counting compared to resting, also EEG spectral power was higher during breath counting compared to resting, albeit not significantly.

Results in sLORETA-based intracortical lagged coherence and Head-surface conventional coherence showed contrary results in that the four significant differences of intracortical lagged coherence between ROIs were lower during breath counting, but the four significant differences of head-surface conventional coherence were higher.

We checked whether the significantly different coherences between ROIs (sLORETA-based intracortical lagged coherence) likewise exhibited contrary results between the corresponding head-surface sites, and whether the significantly different coherences between head-surface sites (Head-surface conventional coherence) likewise exhibited contrary results between the corresponding ROIs. This was true as shown in Table 2B where one case for sLORETA-based intracortical lagged coherence as well as one case for head-surface conventional coherence was significantly (*p* < 0.05) different between conditions but with inverted directions.

## Discussion

As hypothesized, breath counting compared to no-task resting in our meditation-unaware and non-trained participants resulted in decreased brain functional connectivity. Significant changes occurred in specific frequency bands and brain regions. The chosen analysis approach was crucial: Significant changes of functional connectivity during breath counting were reductions when analyzed as intracortical lagged coherence but increases when analyzed as conventional head-surface coherence. Lowered statistical thresholding yielded the same result pattern.

Lowered intracortical lagged coherence during meditation compared to resting has also been observed in experienced meditators (Lehmann et al., 2012). Our present results show that these functional connectivity decreases do not depend on meditation experience and associated trait changes, and not on the awareness that one is executing a meditative exercise. Instead, assuming that breath counting is inductive of a meditative state, our present results suggest that the lowered interactions in neural networks represent a neural mechanism of meditating as such.

The results in Lehmann et al. (2012) Table 3 show mean magnitude of coherence differences between averaged resting and meditation for theta, alpha 2, and beta 3 that are comparable (varying either way by a factor of 2–3) to the presently (Table 2) observed differences of the significant cases. In that study with experienced meditators, the number of significant cases was much larger than in our present study. Presumably, with increased meditation experience, observed coherence decreases become more wide-spread. However, this conclusion remains ambiguous because effects of experience-associated trait changes are convoluted with meditation-induced state changes. Longitudinal designs are needed for clarifying these factors.

The changes of intracortical lagged coherence that we observed occurred in inhibitory, routine function, and facilitatory EEG frequency bands i.e., in slow, medium, and fast frequency bands (Makeig and Jung, 1995; Niedermeyer and Lopes Da Silva, 2005; O'Gorman et al., 2013). The localizations suggest a reduction of frontal BA 10's integrating and control functions (Mölle et al., 1995; Burgess and Ali, 2002; Williams et al., 2005) on right-hemispheric visual imagery functions (BA 17, Kosslyn et al., 1993, 1997), on body scheme functions (BA 40, Bundick and Spinella, 2000; Blanke et al., 2002; Farrer and Frith, 2002), and on somatosensory functions (BA 3). Thereby, local specific processes conceivably are released from the interference by higher-level cognition so that the basic perception of breath counting can remain in the foreground. The topography of the brain regions involved in the lower coherences thus reflects the aim of the breath counting task, i.e., to focus attention on basic bodily perceptions while de-emphasizing effects of mental reasoning. This is to be expected since differences in meditation techniques led to technique-specific localizations of reduced connectivity between the five groups in the Lehmann et al. (2012) study.

Other work that compared EEG connectivity during meditation and resting was done using head-surface coherence. The majority of these publications investigated Transcendental Meditation. In Transcendental Meditation, higher alpha frequency band coherence was observed (Levine, 1976; Dillbeck and Bronson, 1981; Travis and Wallace, 1999; Travis, 2001). Transcendental Meditation, however, explicitly excludes breath counting exercises and excludes attention to bodily sensations in general. Therefore, a comparison with our breath counting results would not be conclusive. Other work investigated Sahaja Yoga meditators where theta frequency band coherence was higher between some and lower between other head surface areas (Aftanas and Golocheikine, 2001). Our results suggest that these findings have to be interpreted with caution as they were observed with an analysis approach that is sensitive to absolute source strength and does not account for ambiguity of localization, reference dependence, and overestimation due to volume conduction.

In line with previous findings (Lehmann et al., 2006), the present results suggest that there need not be a relation between head-surface conventional coherence and intracortical lagged coherence. Hence, comparing head-surface EEG coherence-based reports with our intracortical lagged coherence results is not possible. Both the computation of lagged as opposed to conventional coherence and the computation of intracortical as opposed to head-surface coherences can be expected to contribute to differing results. In fact, it has been shown that an increase in source strength (which is by definition not an increase in coherence) will produce an increase in head-surface EEG conventional coherence which is artificially elevated because of volume conduction (Pascual-Marqui, 1993). However, there is no physics law that relates source strength and lagged intracortical source coherence which is influenced by physiological connections (Pascual-Marqui, 2007; Pascual-Marqui et al., 2011). We note that in the present results there were no significant differences in head surface power (except for a single one of 464 tests) and in sLORETA current density (152 tests) between resting and breath counting.

It can be hypothesized that changes in source strength below significance threshold might suffice to induce corresponding conventional head-surface coherence increases or decreases. However, this cannot be stated conclusively. The differences in conventional head-surface coherence can be caused by a number of additional factors beyond an increase in source strength. For example, a simple minute shift of the cortical generators, without change of strength, from gyrus to sulcus can suffice to lead to a coherence increase between EEG signals. What we would like to point out in the present paper is that when changes in conventional head-surface coherence are reported one simply cannot know whether they originated from truly increased functional connectivity between brain areas and that the results obtained with this measure can be strikingly different from those obtained with measures that address these problems. Thus, an interdependency of head-surface conventional coherence and source strength might also have a role in EEG findings on meditation which suggested that both power and coherence are increased during meditation in the theta and alpha EEG frequency bands (Cahn and Polich, 2006, pp. 181 and 187, respectively).

Our observed intracortical lagged coherences were much smaller than the head-surface conventional coherences. The ranges of our coherence values were comparable to those of similar measures reported in the literature (Travis and Orme-Johnson, 1989). The approach of lagged coherence may omit some true functional connectivity of zero phase angle that can be produced, for instance, by a common, not-recorded source that is simultaneously driving the two ROIs, but the exclusion of zero phase angle values has the advantage that it surely excludes the ubiquitous and large effects of volume conduction that are functionally meaningless (Ruchkin, 2005).

A limitation of our study is that our results were obtained from male participants only. Future work must establish whether women show similar results. We also note that our breath counting instructions did not specify which aspect of breathing should be attended to because we did not want to restrict the spontaneous inclinations of the participants on how to observe their breathing. It remains to be determined whether more restrictive instructions would affect the results. Another limitation is that it cannot be excluded that counting influenced the results. However, we are not aware of papers on brain functional connectivity during counting. Moreover, even if data on counting were available, to compare our results with those found during counting of external events would be difficult because the former activate sensory input and recognition systems and lack the automatic feedback effect on the counted percept which is particular to counting one's own breath.

In conclusion, our study showed that during meditation-type breath counting compared to resting, intracortical lagged coherence exhibited lowered functional connectivity. The observed reductions of functional connectivity were similar but not as widespread as the reductions reported during meditation in experienced meditators (Lehmann et al., 2012). The brain areas involved in decreased functional connectivity during breath counting concerned cognitive control areas and sensory perception areas, conceivably reflecting the goal of breath counting meditation, i.e., to maintain attention on a bodily percept without cognitive reasoning.

### Conflict of interest statement

The authors declare that the research was conducted in the absence of any commercial or financial relationships that could be construed as a potential conflict of interest.
